# Oscillating Laser Conduction Joining of Dissimilar PET to Stainless Steel

**DOI:** 10.3390/polym14224956

**Published:** 2022-11-16

**Authors:** Wei Liao, Suning Zhao, Ming Gao

**Affiliations:** Wuhan National Laboratory for Optoelectronics (WNLO), Huazhong University of Science and Technology, Wuhan 430074, China

**Keywords:** laser joining, polymer–metal joint, beam oscillation, bubble, interface

## Abstract

How to improve the bonding strength of polymers to metals has been one of the challenges in joining fields. It is generally assumed that laser transmission joining is better than laser conduction joining (LCJ) for transparent polymers, and few studies have been focused on LCJ. However, by introducing beam oscillation, an excellent result was obtained in the LCJ of transparent polyethylene terephthalate (PET) to 304 stainless steel. The interface defects of thermal decomposition and bubbles could be eliminated or reduced more efficiently in oscillating laser conduction joining (O-LCJ) rather than transmission joining. Correspondingly, the tensile shear force of joint O-LCJ could be increased by 23.8%, and the plasticity characterized by tensile displacement could be increased by seven times. The improvement mechanism was attributed to two factors by calculating the interface energy distribution and analyzing the force state at the interface. One is the homogenization of interface energy distribution caused by beam oscillation, which decreases the degradation and destruction of polymer macromolecular chains induced by high temperature. The other is the formation of interface bi-directional forces that both inhibit the porosity formation and intensify the chemical reactions. The results bring new insights and provide a new pathway to improve the joining performances of dissimilar polymers to metals.

## 1. Introduction 

Polymers are gradually replacing certain metal parts in automotive, aerospace, and biomedical fields with the advantages of low density, good formability, and low price [1,2,3]. For example, PET polymers are commonly used to make human implants in the biomedical field to replace certain metal parts because of their friction resistance, fatigue resistance, good dimensional stability, and biocompatibility [4,5]. The cross-application of polymer and metallic materials has created a demand for composite joints between them. However, the large difference in physical and chemical properties between polymers and metals makes it difficult to form high-quality composite joints. At present, polymer–metal dissimilar joining methods mainly include chemical gluing, mechanical fastening, and thermal bonding [2,6,7]. Among these, chemical glue joints are less adaptable to the environment, and mechanical fastening joints are insufficiently sealed. Relatively, laser joining as one of the thermal joining has the advantages of high speed, no contact, and easy automation [8,9], which makes high-quality polymer–metal composite joints possible.

In general, the laser joining of polymers to metals can be divided into two types: laser transmission joining (LTJ) and laser conduction joining (LCJ) [1]. In LTJ, the laser directly acts on the joining interface to melt the polymer to form a joint with the metal, which is mainly used for plastics with high transparency, including polyethylene terephthalate (PET), polymethyl methacrylate (PMMA), polycarbonate (PC), polyethylene (PE), and acrylonitrile (ABS) [10,11,12,13,14]. In LCJ, the laser energy is absorbed by the upper layer of metal and then transferred to the interface to establish a joint, and the method is applicable to polymers of any transparency. In both laser transmission and conduction joining, the concentration of laser energy brings about high temperature gradients, resulting in uneven joint morphology. For example, in a study by Chan et al. [12] on LTJ of PET and pure titanium, it was found that the PET in the middle of the joint showed high-temperature decomposition discoloration, and the bond strength of the joint was severely reduced when the percentage of discolored area was higher than 30%. Moreover, the result of Xue et al. [15] showed that the joint area in PA6GF30 and SUS444 LCJ could be divided into a decomposition area and a non-decomposition area, and the larger the non-decomposition area, the better the mechanical properties of the joint.

Compared with LTJ, the laser heat source of LCJ cannot act on the interface directly [16]. This makes the interface temperature monitoring and energy regulation more difficult in LCJ and further limits its application in transparent polymers. Some scholars have studied the influence of lap configuration on joint quality [17,18,19,20] and found that with LCJ it is difficult to obtain higher joint quality under Gaussian spots. Hussein et al. [20] found that LCJ was more sensitive to heat input in the joint of PMMA and 304 stainless steel (304SS) and only obtained a highest joint tensile force of 495 N, 54% of that of LTJ. Huang et al. [8] found that the heat input must be controlled below 3.92 J/mm^2^ to avoid the thermal deterioration defects in LCJ between PMMA and 304SS, but low heat input cannot guarantee the joint tensile force. In LCJ of 304SS and ABS, Peng et al. [21] found that the interface bubbles and process instability increased significantly when the temperature increased from 120 to 180 °C. This indicated that the appropriate joining temperature range of thermoplastic polymers should be between the melting and decomposition temperatures [1,22], but it is difficult to be controlled because the range is only a few tens of degrees. However, it is noticeable that a few researchers have obtained the converse results. For example, Wahba et al. [19] obtained a higher strength of PET/Mg joints via LCJ rather than LTJ when using rectangular spot laser joining, which showed the possibility of using LCJ to improve the joint strength for transparent polymer homogenizing laser power distribution.

On the other hand, the joint interface force state of LTJ and LCJ is different during joining. In order to ensure the necessary reaction force of polymer–metal joints, André et al. [23] proposed to use glass clamping in LTJ and mask clamping in LCJ. Some research showed the effects of interface force on the properties of polymer–metal joints. Jiao et al. [24] found that the joint strength was increased and then decreased with the increase of fixture pressure in dissimilar CFRTP and stainless-steel joints. In addition, Hossein et al. [25,26,27,28] studied the force state and failure behavior of fiber-reinforced polymer (FRP) tubular joints and optimized the FRP layer number and joint geometry by simulation. Rajak et al. [29,30] discussed the force models of FRP joints with different cross-sectional shapes, which could improve the tubular joint design in practical applications. All the research studies above demonstrated that the joint force state would play a big role in changing the quality of polymer–metal joints. Let us return to Wahba’s study [19] discussed above to ask the question, “did using a rectangular spot laser not only homogenize the power distribution but also induce some advantages in the joint force state of LCJ”?

In recent years, laser oscillation welding has allowed more precise control of the laser energy distribution by adjusting the oscillation frequency and amplitude [31,32,33,34], which can obtain better process stability and welding performance without any pretreatment. This property has been well demonstrated in the field of welding of homogeneous and dissimilar materials. For example, Ke et al. [31] found that beam oscillation increases the melt pool width for laser welding of aluminum alloys and stirs the keyhole in the melt pool to promote bubble overflow. Jiao et al. [32] found that bubble defects in CFRTP and aluminum alloy joints could be reduced by high-speed rotational welding. In addition, the research of Shi et al. [33] demonstrated that beam oscillation created a stirring effect to promote solute flow and uniformize the temperature distribution of the molten pool in laser welding of aluminum alloys. Meng et al. [34] found that beam oscillation could promote the element distribution uniformity in laser welding of aluminum/steel dissimilar materials, thus nearly doubling the tensile strength of joints. More importantly, our previous research found that the use of beam oscillation in LTJ of PET and 304SS can improve the thermal deterioration defects of the joint by optimizing the scanning radius, which is 26% higher than the shear tension of the non-scanned joint [35].

For LCJ of transparent polymers, the laser action position and the force state at the interface are changed in comparison of LTJ. Which obtains better effects in LCJ when beam oscillation is employed? Less research has been addressed on this topic so far. This study therefore carried out experiments of the oscillating laser joining of transparent PET and 304SS and discussed the strength improvement mechanisms on the basis of laser beam energy distribution and the interface force. 

## 2. Materials and Methods

### 2.1. Experimental Equipment and Materials

**Figure 1:**
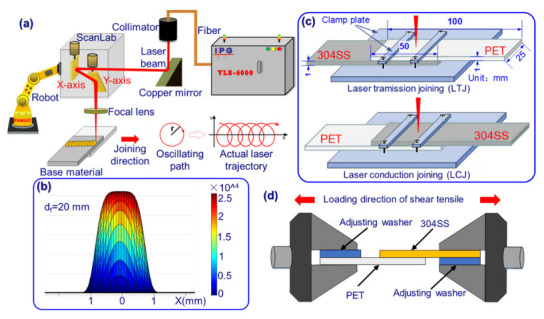
(**a**) Schematic diagram of experimental equipment, (**b**) energy distribution of spot at 20 mm of focus position, (**c**) clamping configurations corresponding to the two joining methods, (**d**) schematic diagram of composite joint tensile-shear test.

The experimental setup was composed of a fiber laser (wavelength: 1064 nm), a robot, and a galvanometer welding head, as shown in Figure 1. The galvanometer was used to scan the laser in a circle, and the actual beam trajectory after superimposing the linear motion of the robot is shown in Figure 1a. The defocus during joining was fixed at 20 mm, and the energy distribution at the spot is shown in Figure 1b. Two lap configurations of LTJ and LCJ were designed, as shown in Figure 1c. The experimental base materials were 304SS (chemical composition: Fe-18.09Cr-8.01Ni-1.25Mn wt%) and transparent PET with a size of 100 mm × 25 mm × 1 mm. The physical properties of PET are shown in Table 1. The joining parameters are shown in Table 2, where scanning parameters (r = 2 mm, f = 300 Hz) are the optimal parameters obtained after LTJ optimization [35].

### 2.2. Experimental Methods

Acetone was used to remove oil stains on the surface of 304SS before joining. A Zeiss stereo microscope was used to observe the macroscopic morphology of joints. The tensile shear test (as shown in Figure 1d) was carried out according to ISO 4136:2001, and the average value of the same parameters was obtained by repeating the test three times. Tensile displacement was discussed for the first time in this study to characterize the joint toughness, which is defined as the displacement between PET and 304SS from the beginning of stretching to the time of joint fracture. Scanning electron microscopy (SEM) and X-ray photoelectron spectroscopy (XPS) were used to analyze the joint morphology and element morphology of the fractured samples. Before XPS detection, the sample surface was sputtered with an Ar-ion beam (500 eV) for 100 s to remove surface contaminants from any unknown source.

## 3. Results

### 3.1. Joint Morphology Characteristics

**Figure 2:**
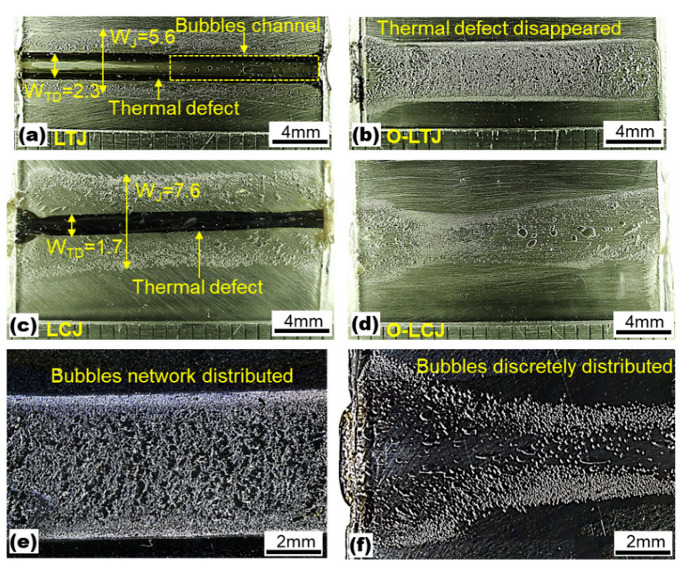
Joint macroscopic morphologies: (**a**) laser transmission joining (LTJ), (**b**) oscillating laser transmission joining (O-LTJ), (**c**) laser conduction joining (LCJ), (**d**) oscillating laser conduction joining (O-LCJ), (**e**) magnification of O-LTJ, (**f**) magnification of O-LCJ.

The macroscopic morphologies of joints are shown in Figure 2. The results of Figure 2a,c show that there were obvious thermal deterioration defects in joints without oscillation. Among them, the width (W_J_) of joint LTJ was 5.6 mm, and the width of the thermal deterioration defect (W_TD_) was 2.3 mm, as shown in Figure 2a. In addition, a series of bubble channels appeared inside the joint. The width of joint LCJ and the thermal deterioration defect were 7.6 mm and 1.7 mm, respectively, as shown in Figure 2c. Under the same thermal input, the joint width of LCJ was 35.7% higher than that of LTJ. After using beam oscillation, the thermal deterioration defects in LTJ and LCJ were eliminated successfully, and the joint morphology uniformity was improved, as shown in Figure 2b,d. Under the same joining parameters, the bubbles in the oscillating laser transmission joining (O-LTJ) were interconnected in a network distribution, as shown in Figure 2e, while the bubbles in the oscillating laser conduction joining (O-LCJ) were discretely distributed with a reduced number, as shown in Figure 2f.

**Figure 3:**
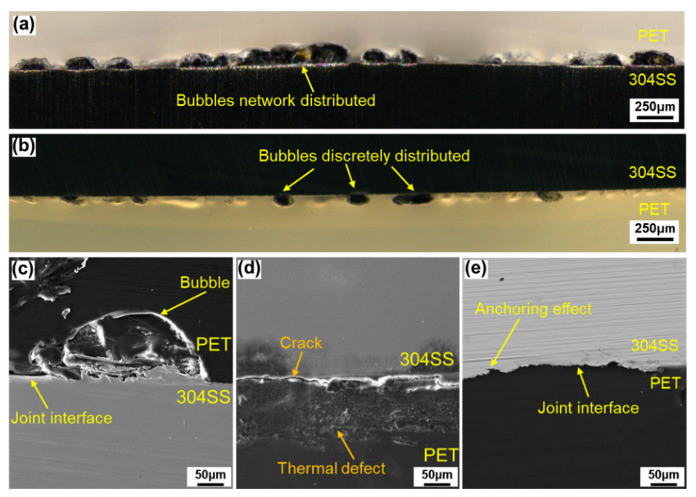
Cross-section of joints: (**a**) bubble distribution of O-LTJ, (**b**) bubble distribution of O-LCJ, (**c**) bubble detail of O-LTJ, (**d**) thermal defect and crack of LCJ, (**e**) joining region of O-LCJ.

The cross-section morphologies of the joint are shown in Figure 3. It can be seen from Figure 3a that a large number of bubbles fused with each other and presented a chain distribution in the O-LTJ joint, which conformed to the network distribution characteristics of Figure 2e. Compared with O-LTJ, the bubbles in O-LCJ were independent of each other and presented a discrete distribution, as shown in Figure 3b, and the result is consistent with Figure 2f. The magnified view of the O-LTJ joint shows no significant change in 304SS, while the PET of the interface formed large bubbles due to decomposition, as shown in Figure 3c. However, the morphology in Figure 3d shows a rough surface with many micro-bubbles and micro-cracks occurring in the PET of the LCJ joint, and there are also obvious cracks in the interface. The interface cracks were mainly caused by the solidification and shrinkage of PET after melting and decomposing at high temperatures [36]. As can be seen in Figure 3e, the molten PET at the interface filled the crater on the surface of 304SS to form a mechanical anchorage in the non-bubble area of the O-LCJ joint.

### 3.2. Tensile Properties and Failure Characterization

**Figure 4:**
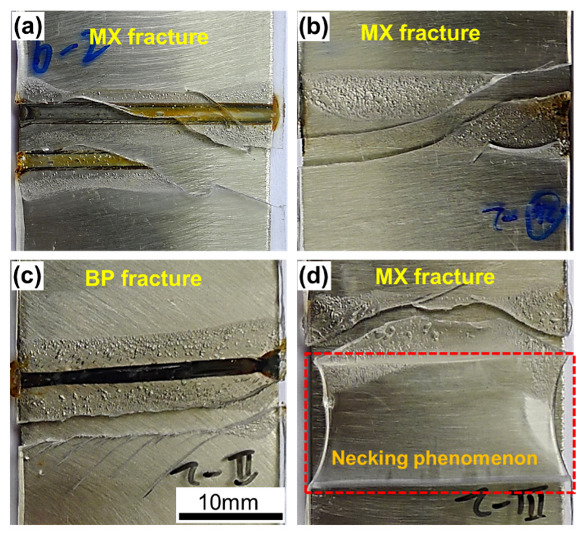
Macroscopic fracture morphologies: (**a**) LTJ, (**b**) O-LTJ, (**c**) LCJ, (**d**) O-LCJ, where “MX fracture” denotes the sample cracks in mixed mode; “BP fracture” denotes the sample cracks along PET.

**Figure 5:**
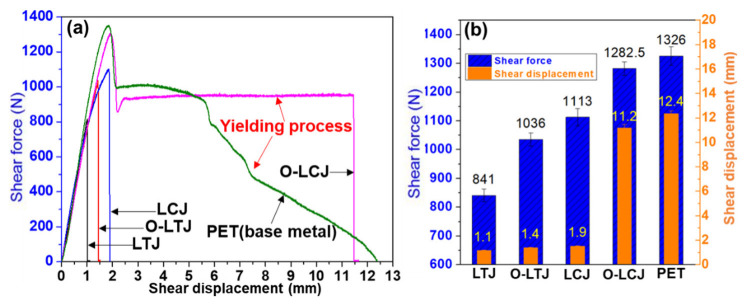
(**a**) Tensile curves (each parameter shows a sample); (**b**) tensile properties of the joints.

The tensile fracture morphologies and mechanical properties of joints are shown in Figure 4 and Figure 5, respectively. As shown in Figure 4a, joint LTJ showed a mixed fracture mode at the interface with an average shear tensile force of 841.2 N and a displacement of 1.1 mm. However, the shear tensile force and displacement of joint O-LTJ were increased by 26% and 27%, respectively. The joint LCJ fractured at the PET rather than at the interface, as shown in Figure 4c, and had a higher shear tensile force of 1113.3 N and a displacement of 1.9 mm. After using beam oscillation, the shear tensile force and displacement of joint O-LCJ were further increased to 1282.5 N and 11.5 mm respectively, 96.7% and 90% of PET, respectively. In addition, as shown in Figure 4d, an obvious necking occurred, corresponding to the yield process in the tensile curve in Figure 5a, which was similar to PET. Compared to O-LTJ, joint O-LCJ had a higher tensile force increased by 23.8%, and a larger displacement increased by 7 times. This suggests that the discrete distribution of interface bubbles of joint O-LCJ is beneficial to the performance, which is better than the network distribution of joint O-LTJ. Moreover, the O-LCJ joint had better plasticity due to the obviously increased displacement.

**Figure 6:**
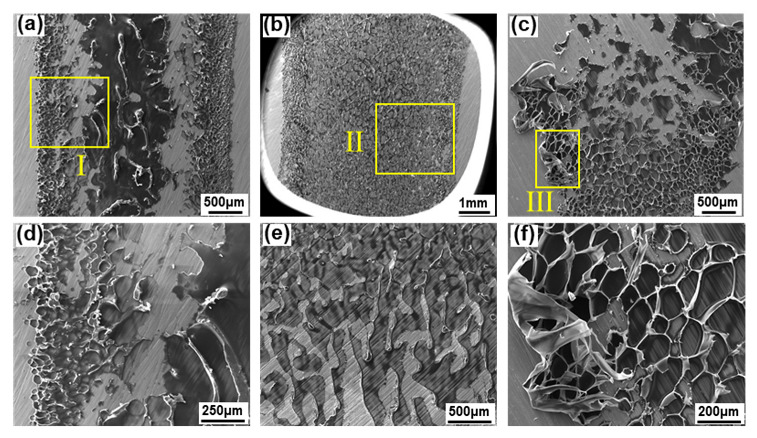
Fracture surfaces at 304SS side of tensile samples: (**a**) LTJ, (**b**) O-LTJ, (**c**) O-LCJ, (**d**) details of region I, (**e**) details of region II, (**f**) details of region III.

The microfracture morphologies of the 304SS side were observed by SEM in order to analyze the fracture surface characteristics. As shown in Figure 6a, the residual PET in the LTJ joint showed two morphologies; one was the middle area with a thicker residual layer of more serious damage, and the other was the area of bubbles on both sides with a thinner residual layer. Both morphologies showed an obvious brittle fracture, as shown in Figure 6d. Although the surface also showed a brittle fracture in the O-LTJ joint, the residual PET was uniform and smooth, as shown in Figure 6b,e. Unlike the smooth surface of O-LTJ, the fracture surface of the O-LCJ joint was rough, with obvious plastic deformation, as shown in Figure 6c,f, mainly showing ductile fracture characteristics.

**Figure 7:**
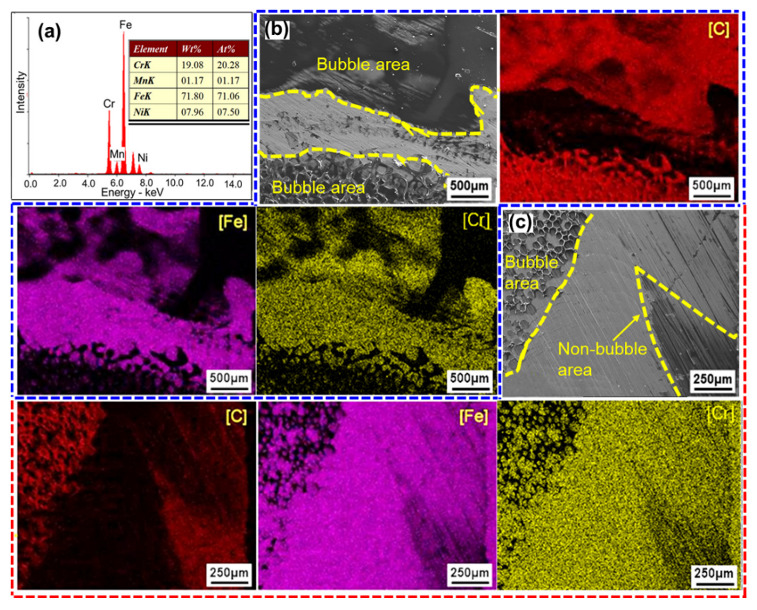
Chemical composition at the 304SS side of the fractured surface: (**a**) EDS of 304, (**b**) EDS mapping of LTJ, (**c**) EDS mapping of O-LCJ.

As shown in Figure 7a, the base metal 304SS mainly contained the elements of Fe, Cr, and Ni. In joint LTJ, the C-rich region was found in the bubble areas, and only a small amount of C was detected in the non-bubble areas. The characterizations of Fe and Cr were complementary to those of C, the high concentration of which mainly appeared in the non-bubble areas, as shown in Figure 7b. In O-LCJ, the C-rich region both occurred in the bubble areas and non-bubble areas, as shown in Figure 7c, indicating a higher bonding strength of non-bubble areas in O-LCJ. No matter LTJ or O-LCJ, the residues of C, Fe, and Cr appeared in the non-bubble areas at the same time, indicating that some chemical reactions occurred at the interface.

### 3.3. Analysis of XPS Results

Numerous studies have shown that mechanical anchoring, chemical bonding, electronic interaction, diffusion, and adsorption are the five main bonding mechanisms at the interface of polymer–metal joints [37,38,39,40,41], among which XPS is an important means to study the chemical bonding mechanism of the joint interface. For example, Chan et al. [12] found that Ti–C chemical bonds played an important contribution to the high strength of the joint in laser joining of PET to pure titanium by XPS detection. In addition, Liu et al. [41] found that carbonyl components (C=O) in polymers provided high activity, and it was easy to form C–O–M bonds with metal at the interface.

**Figure 8:**
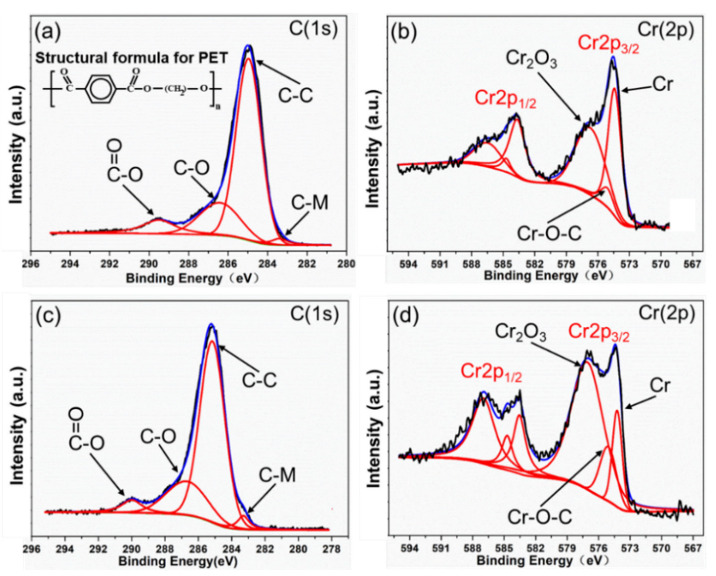
The XPS narrow scan spectrum of C and Cr elements: (**a**) C(1s) spectrum of O-LTJ, (**b**) Cr(2p) spectrum of O-LTJ, (**c**) C(1s) spectrum of O-LCJ, (**d**) Cr(2p) spectrum of O-LCJ.

Based on the above theory, the elemental spectrum of the interfaces of O-LCJ and O-LTJ were analyzed by XPS, respectively. As Figure 8a,c shows, the split-peak fitting showed that in addition to three peaks in the C(1s) spectrum at 284.8 eV (C–C), 286.5 eV (C–O), and 289.2 eV (O–C=O) (the split-peak positions are based on Ref. [40]), there was also a peak at the binding energy 283.4 eV position. The binding energy at position 283.4 eV in the C(1s) spectrum was presumed to correspond to the C–M chemical bond based on the results of Refs. [38,42]. The results of the Cr(2p) spectrum split-peak fitting were 574.2 eV (Cr), 575.1 eV (C–O–Cr), and 576.2 eV (Cr_2_O_3_), as shown in Figure 8b,d. The split-peak positions were mainly based on Ref. [38]. Among them, the C–M and C–O–Cr bonds were newly formed due to the chemical reaction between PET and 304SS. As shown in Table 3, the relative content (the ratio of single peak area to the total area) of O=C–O bonds decreased from 9.5% to 5.4% in O-LCJ, while the relative content of C–M increased from 2.5% to 3.6%. In addition, the relative content of Cr decreased from 45% to 14.3%, while the content of newly formed C–O–Cr bonds increased from 7.7% to 14.3%. This suggests that more C–M and C–O–Cr bonds appeared in the O-LCJ joint, which was one of the reasons for the improvement of its tensile properties.

## 4. Discussion

### 4.1. Energy Distribution Model

Heat conduction is the main form of heat transfer in laser joining between polymer and metal, which realizes heat transfer through the thermal motion of optical particles in contact with discrete points. The coefficient of heat conductivity (*h_c_*) is calculated by Equation (1) [43,44]:

(1) $$h_{c}=1.25K_{s}\sqrt{R_{a1}^{2}+R_{a2}^{2}}\left(\frac{p}{H_{c}}\right)$$

where *K_s_* is the heat conductivity of phonons, and *R_a1_* and *R_a2_* are the roughness of polymer and metal at the interface, respectively. *P* is the stress of the interface, and *H_c_* is the hardness of the polymer.

The parameters *K_s_*, *R_a1_*, *R_a2_*, and *H_c_* are only related to the properties of the material itself, and the parameter *p* is related to the joining state. In LTJ, the interface is only subjected to the unidirectional force provided by 304SS. However, in LCJ, the interface is subjected to bi-directional forces provided by both the 304SS and the fixture. Therefore, LCJ has a larger *p* and a higher *h_c_* compared with LTJ. In addition, because the laser transmittance of the PET is only 90% [13], there is a 10% energy loss in the transmission joining interface. What is more, the interface temperature during the laser joining of polymer to metal does not exceed 500 °C [11], resulting in a small thermal conductivity (around 16 W/(m·K)) of 304SS, which may be more favorable to LCJ. For these reasons, the LCJ has higher energy transfer efficiency than the LTJ, although the distance between the laser and the joining interface is larger. Therefore, the width of joint LCJ is 35.7% higher than that of LTJ under the same joining parameters.

The energy distribution of the beam oscillation in the joint is calculated based on the energy deposition model proposed by Mahrle et al. [45]. Firstly, the laser spot energy distribution is calculated by substituting laser beam quality and process parameters into the standard Gaussian distribution model. Secondly, the computed energy distribution is substituted into the circular oscillation equation. Finally, the interface energy distribution results of laser oscillation are shown in a three-dimensional picture using MATLAB by periodic integration operations.

**Figure 9:**
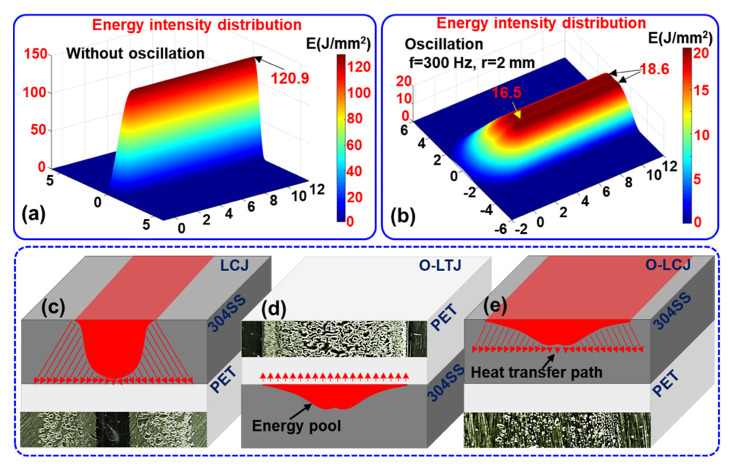
The energy intensity distribution and heat transfer model of joints: (**a**) energy intensity distribution without oscillation, (**b**) energy intensity distribution with oscillation, (**c**) heat transfer model of LCJ, (**d**) heat transfer model of O-LTJ, (**e**) heat transfer model of O-LCJ.

As shown in Figure 9a, the energy distribution at the joint interface is very concentrated and presented in the form of a single wave peak when it is without oscillation. The energy density in the center of the joint reaches 120.9 J/mm^2^ with a large attenuation gradient to both sides. After oscillating with a circular beam of 2 mm amplitude and 300 Hz frequency, the energy distribution in the joint is more uniform and shows a double wave peak form with two high sides and a low middle. The energy density of the wave peaks and middle trough are 18.6 J/mm^2^ and 16.5 J/mm^2^, respectively. The energy attenuation gradient to both sides is small, as shown in Figure 9b. Since the LCJ is not oscillating, the energy in the center of the joint is very concentrated according to the energy distribution model, which leads to serious thermal deterioration defects of the PET in the center of the joint, as shown in Figure 9c. Moreover, the thermal deterioration in the central area of the joint in LCJ proved that the temperature exceeded the decomposition temperature of PET. The high temperature led to a sudden change in the crystallinity of the polymers [14], which reduced the plasticity of the PET. Therefore, brittle fractures of PET occurred during the tensile process of the LCJ joint. After using beam oscillation, the uniformity of energy distribution is improved significantly in O-LTJ, and the heat is transferred over equal distances. The PET at the interface is heated more uniformly and the bubbles are of the same size, as shown in Figure 9d. In O-LCJ, the energy distribution uniformity is the same as in O-LTJ, but the energy transfer direction is opposite, and the transfer distance is increased, as shown in Figure 9e. The joint interface temperature decreases as the heat transfer distance increases, but the heat range also increases at the same time, which helps to further reduce the energy gradient at the interface, thus improving the morphology of O-LCJ.

### 4.2. Effect of Interface Forces on Joint Morphology

**Figure 10:**
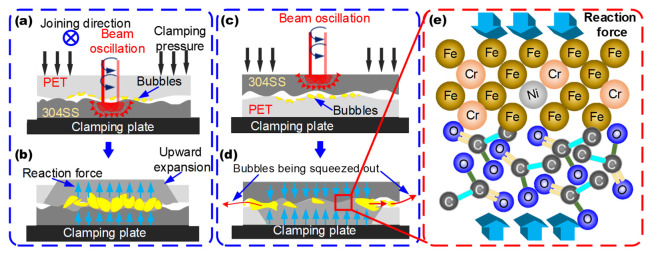
Formation of bubbles and chemical reaction of the interface: (**a**) joining process diagram of O-LTJ, (**b**) bubble force analysis of O-LTJ, (**c**) joining process diagram of O-LCJ, (**d**) bubble force analysis of O-LCJ, (**e**) chemical reaction mechanism of the interface.

In order to further explore the joint morphology mechanism, the force models of the interface of O-LTJ and O-LCJ are established, respectively, as shown in Figure 10. In O-LTJ, the laser penetrating PET heats 304SS to raise the interface temperature, and the PET at the interface is decomposed by heat and begins to form bubbles, which continue to expand as the temperature rises, as shown in Figure 10a. When the temperature reaches *T_f_*, the interaction forces between the molecular chains of the PET in the viscous flow state have been disrupted [46]. The macromolecular chain breaks and relative displacement occurs, which is evidenced by the expansion and deformation of the polymers, as shown in Figure 10b. Therefore, the bubbles are only subjected to the single upward reaction force provided by 304SS, which causes them to expand toward the PET interior, as shown in the yellow area of Figure 10b. The bubbles gradually form a network distribution after growth, expansion, and fusion. 

In O-LCJ, the laser heats 304SS directly and transfers the heat to the joint interface, where the PET is decomposed to form bubbles, as shown in Figure 10c. Since 304SS has a much higher elastic modulus (*E*: about 200 GPa) than PET (*E*: about 4 GP) and its thermal deformation temperature is in the range of 900–1150 °C [47], the interface temperature is much lower than its deformation temperature, so sufficient stiffness is always maintained during the joining. Therefore, the 304SS in the upper layer and the clamps in the lower layer in O-LCJ provide bi-directional forces for the reactive interface, limiting the pathway for the growth and fusion of bubbles into the PET interior. On the other hand, the circular motion of the beam oscillation will form a stirring effect in the PET molten pool [24], which interacts with the bi-directional force to cause some of the bubbles to be squeezed out from the interface gap (as shown by the red arrows in Figure 10d). Finally, the discrete distribution is obtained by reducing the number of bubbles. In addition, the bi-directional squeezing force can provide a greater reaction pressure for the chemical reaction between the PET and 304SS interface, which promotes the formation of C–M and C–O–Cr chemical bonds, as shown in Figure 10e.

## 5. Conclusions 

The major conclusions are summarized as follows:

(1) Joint morphology was improved after introducing beam oscillation; the bubbles in O-LTJ showed a network distribution, and the bubbles in O-LCJ mainly showed discrete distributions with a reduced number.
(2) The joint shearing force of O-LCJ was increased by 23.8%, and the tensile displacement was increased by seven times compared with O-LTJ. The results showed that O-LCJ could obtain higher-quality polymer–metal composite joints than O-LTJ.
(3) Beam oscillation can reduce the interfacial energy gradient, which helps to mitigate the degradation and destruction of polymer macromolecular chains induced by high temperature.
(4) The cooperation between bi-directional squeezing force and beam oscillation is the main reason for performance improvement, which inhibited the growth and fusion of bubbles and promoted some bubbles to escape from the interface gap, thus obtaining a discrete distribution of the bubbles by reducing their number.

The results may help to solve problems in polymer–metal composite joints and broaden their application scope and may also provide new guidelines for the design and manufacture of polymer–metal composite joints.

## Figures and Tables

**Table 1:** Physical properties of PET.

Physical Properties	Glass Transition Temperature (*T_g_*)	Flow Temperature (*T_f_*)	Decomposition Temperature (*T_d_*)	Light Transmittance	Relative Density
Value	68–80 °C	212–265 °C	280–370 °C	90%	1.38

**Table 2:** Joining process variables.

Joint	Oscillating Radius, r (mm)	Oscillating Frequency, *f* (Hz)	Fixed Parameters
LTJ	0	0	Laser power (P) = 300 W Joining speed (V) = 50 cm/min Focus position (d_f_) = 20 mm
O-LTJ	2	300	
LCJ	0	0	
O-LCJ	2	300	

**Table 3:** Percentage of atoms with different chemical bonds after split-peak fitting of C(1s) spectra and Cr(2p) spectra.

Spectra	Chemical Bonds	Binding Energy (eV)	Atomic Percentage of O-LTJ Joint (%)	Atomic Percentage of O-LCJ Joint (%)
C(1s)	C–C	284.8	68.1	69.7
	C–O	286.5	19.9	21.2
	O–C=O	289.2	9.5	5.4
	C–M	283.4	2.5	3.6
Cr(2p)	Cr–O–C	574.2	7.7	14.3
	Cr_2_O_3_	575.1	47.3	72.3
	Cr	576.2	45	14.3

## Data Availability

The data presented in this study are available upon request from the corresponding author.

## Notation List

PET	Polyethylene terephthalate
LTJ	Laser transmission joining
O-LTJ	Oscillating laser transmission joining
LCJ	Laser conduction joining
O-LCJ	Oscillating laser conduction joining
PC	Polycarbonate
PE	Polyethylene
ABS	Acrylonitrile
PMMA	Polymethyl methacrylate
PA6GF30	Polyamide 6 with 30% glass fiber
FRP	Fiber-reinforced polymer
CFRTP	Carbon fiber-reinforced thermal plastic
SUS444	444 stainless-steel
304SS	304 stainless-steel
*T_f_*	Flow temperature

## References

[1] Huang Y., Gao X., Zhang Y., Ma B. (2022). Laser joining technology of polymer-metal hybrid structures—A review. J. Manuf. Proc..

[2] Kah P., Suoranta R., Martikainen J., Magnus C. (2014). Techniques for joining dissimilar materials: Metals and polymers. Rev. Adv. Mater. Sci..

[3] Sultana T., Georgiev G., Baird R., Auner G., Newaz G., Patwa R., Herfurth H. (2009). Study of two different thin film coating methods in transmission laser micro-joining of thin Ti-film coated glass and polyimide for biomedical applications. J. Mech. Behav. Biomed. Mater..

[4] Jaime S.B.M., Rosa M.V., Alves P., Bocoli F.J. (2022). Moisture and oxygen barrier properties of glass, PET and HDPE bottles for pharma-ceutical products. J. Drug Deliv. Sci. Technol..

[5] Moghadasi K., Isa M.S.M., Arffin M.A., Mohdjamil M.Z., Wu B., Yamani M., Muhamad M.R.B., Yusof F., Jamaludin M.F., bin Ab Karim M.S. (2022). A review on biomedical implant materials and the effect of friction stir based techniques on their mechanical and tribological properties. J. Mater. Res. Technol..

[6] Galińska A., Galiński C. (2020). Mechanical Joining of Fibre Reinforced Polymer Composites to Metals—A Review. Part II: Riveting, Clinching, Non-Adhesive Form-Locked Joints, Pin and Loop Joining. Polymers.

[7] Lambiase F., Scipioni S., Lee C.-J., Ko D.-C., Liu F. (2021). A State-of-the-Art Review on Advanced Joining Processes for Metal-Composite and Metal-Polymer Hybrid Structures. Materials.

[8] Huang Y., Gao X., Ma B., Liu G., Zhang N., Zhang Y., You D. (2020). Optimization of weld strength for laser welding of steel to PMMA using Taguchi design method. Opt. Laser Technol..

[9] Tamrin K.F., Nukman Y., Zakariyah S.S. (2013). Laser Lap Joining of Dissimilar Materials—A Review of Factors Affecting Joint Strength. Mater. Manuf. Proc..

[10] Seiji K., Yousuke K. (2008). Laser direct joining of metal and plastic. Scr. Mater..

[11] Lambiase F., Genna S. (2017). Laser-assisted direct joining of AISI304 stainless steel with polycarbonate sheets: Thermal analysis, mechanical characterization, and bonds morphology. Opt. Laser Technol..

[12] Chan C.W., Graham C.S. (2016). Fibre laser joining of highly dissimilar materials: Commercially pure Ti and PET hybrid joint for medical device applications. Mater. Des..

[13] Ai Y.W., Zheng K., Shin Y.C., Wu B. (2018). Analysis of weld geometry and liquid flow in laser transmission welding between poly-ethylene terephthalate (PET) and Ti6Al4V based on numerical simulation. Opt. Laser Technol..

[14] Xu W., Li P., Liu H., Wang H., Wang X. (2022). Numerical simulation of molten pool formation during laser transmission welding between PET and SUS304. Int. Commun. Heat Mass Transf..

[15] Xue Z., Shen J., Hu S. (2022). Influence of scanning speed and defocus distance on laser welded PA6GF30/SUS444 dissimilar lap joints. Opt. Laser Technol..

[16] Wang Q., Jia Z.-Y., Zhang B.-Y., Fu R., Liu J.-Y., Han D.-Z. (2022). Study on interface temperature control of laser direct joining of CFRTP and aluminum alloy based on staged laser path planning. Opt. Laser Technol..

[17] Fortunato A., Cuccolini G., Ascari A., Orazi L., Campana G., Tani G. (2010). Hybrid metal-plastic joining by means of laser. Int. J. Mater. Form..

[18] Bergmann J.P., Stambke M. (2012). Potential of laser-manufactured polymer-metal hybrid joints. Phys. Procedia.

[19] Wahba M., Kawahito Y., Katayama S. (2011). Laser direct joining of AZ91D thixomolded Mg alloy and amorphous polyethylene terephthalate. J. Mater. Proc. Technol..

[20] Hussein F.I., Akman E., Oztoprak B.G., Gunes M., Gundogdu O., Kacar E., Hajim K., Demir A. (2013). Evaluation of PMMA joining to stainless steel 304 using pulsed Nd:YAG laser. Opt. Laser Technol..

[21] Peng Y., Barzinjy A.A., Al-Rashed A.A., Panjehpour A., Mehrjou M., Afrand M. (2019). Investigation the effect of pulsed laser parameters on the temperature distribution and joint interface properties in dissimilar laser joining of austenitic stainless steel 304 and Acrylonitrile Butadiene Styrene. J. Manuf. Proc..

[22] Lambiase F., Genna S., Kant R. (2018). Optimization of laser-assisted joining through an integrated experimental-simulation ap-proach. Int. J. Adv. Manuf. Technol..

[23] André H., Zaeh M.F. (2014). Laser Surface Pre-treatment of Aluminium for Hybrid Joints with Glass Fibre Reinforced Thermoplastics. Phys. Procedia.

[24] Jiao J., Xu Z., Wang Q., Sheng L., Zhang W. (2018). CFRTP and stainless steel laser joining: Thermal defects analysis and joining parameters optimization. Opt. Laser Technol..

[25] Nassiraei H., Rezadoost P. (2021). Parametric study and formula for SCFs of FRP-strengthened CHS T/Y-joints under out-of-plane bending load. Ocean Eng..

[26] Nassiraei H., Rezadoost P. (2021). Stress concentration factors in tubular T/Y-connections reinforced with FRP under in-plane bending load. Mar. Struct..

[27] Nassiraei H., Rezadoost P. (2021). Local joint flexibility of tubular T/Y-joints retrofitted with GFRP under in-plane bending moment. Mar. Struct..

[28] Nassiraei H., Rezadoost P. (2021). Static capacity of tubular X-joints reinforced with fiber reinforced polymer subjected to compressive load. Eng. Struct..

[29] Rajak D.K., Wagh P.H., Ashwini K., Sanjay R.M., Siengchin S., Khan A., Asiri A.M., Nareh K., Velmurugan E., Gupta N.K. (2022). Impact of fiber reinforced polymer composites on structural joints of tubular sec-tions: A review. Thin-Walled Struct..

[30] Rajak D.K., Wagh P.H., Linul E. (2021). Manufacturing Technologies of Carbon/Glass Fiber-Reinforced Polymer Composites and Their Properties: A Review. Polymers.

[31] Ke W., Bu X., Oliveira J., Xu W., Wang Z., Zeng Z. (2021). Modeling and numerical study of keyhole-induced porosity formation in laser beam oscillating welding of 5A06 aluminum alloy. Opt. Laser Technol..

[32] Jiao J., Ye Y., Jia S., Xu Z., Ouyang W., Zhang W. (2020). CFRTP -Al alloy laser assisted joining with a high speed rotational welding technology. Opt. Laser Technol..

[33] Shi L., Jiang L., Gao M. (2022). Numerical research on molten pool dynamics of oscillating laser-arc hybrid welding. Int. J. Heat Mass Transf..

[34] Meng Y., Gong M., Zhang S., Zhang Y., Gao M. (2020). Effects of oscillating laser offset on microstructure and properties of dissimilar Al/steel butt-joint. Opt. Lasers Eng..

[35] Hao K., Liao W., Zhang T., Gao M. (2020). Interface formation and bonding mechanisms of laser transmission welded composite structure of PET on austenitic steel via beam oscillation. Compos. Struct..

[36] Tan X., Zhang J., Shan J., Yang S., Ren J. (2015). Characteristics and formation mechanism of porosities in CFRP during laser joining of CFRP and steel. Compos. Part B Eng..

[37] Hirchenhahn P., Al Sayyad A., Bardon J., Felten A., Plapper P., Houssiau L. (2020). Highlighting Chemical Bonding between Nylon-6.6 and the Native Oxide from an Aluminum Sheet Assembled by Laser Welding. ACS Appl. Polym. Mater..

[38] Zou X., Chen K., Yao H., Chen C., Lu X., Ding P., Wang M., Hua X., Shan A. (2022). Chemical Reaction and Bonding Mechanism at the Polymer–Metal Interface. ACS Appl. Mater. Interfaces.

[39] Liu J., Zhang Q., Zhang B., Yu M. (2020). The Bonding Mechanism of the Micro-Interface of Polymer Coated Steel. Polymers.

[40] Rouba G., Parinaz S., Rino M., De Geyter N. (2022). Chemical characterization of plasma-activated polymeric surfaces via XPS analyses: A review. Surf. Interfaces.

[41] Liu F., Dong P., Lu W., Sun K. (2019). On formation of Al O C bonds at aluminum/polyamide joint interface. Appl. Surf. Sci..

[42] Tan X., Shan J., Ren J. (2013). Effects of cr plating layer on shear strength and interface bonding characteristics of mild steel/cfrp joint by laser heating. Acta Met. Sin..

[43] Chang D., Wang R. (2012). Thermal contact conductance of stainless steel-GFRP interface under vacuum environment. Exp. Therm. Fluid Sci..

[44] Bahrami M., Yovanovich M.M., Marotta E.E. (2006). Thermal Joint Resistance of Polymer-Metal Rough Interfaces. J. Electron. Packag..

[45] Mahrle A., Beyer E. Modeling and simulation of the energy deposition in laser beam welding with oscillatory beam deflection. Proceedings of the 26th International Congress on Laser Materials Processing.

[46] Wolfgang G., Sabine S., Grellmann W., Seidler S. (2022). Fracture Toughness Measurements in Engineering Polymers. Polymer Testing.

[47] Kori P., Vadavadagi B.H., Khatirkar R.K. (2020). Hot deformation characteristics of ASS-304 austenitic stainless steel by tensile tests. Mater. Today Proc..

